# Influences on Pregnant Women’s and Health Care Professionals’ Behaviour Regarding Maternal Vaccinations: A Qualitative Interview Study

**DOI:** 10.3390/vaccines10010076

**Published:** 2022-01-04

**Authors:** Natalie Gauld, Samuel Martin, Owen Sinclair, Helen Petousis-Harris, Felicity Dumble, Cameron C. Grant

**Affiliations:** 1 Department of Paediatrics: Child and Youth Health, University of Auckland, Auckland 1023, New Zealand; cc.grant@auckland.ac.nz; 2 School of Pharmacy, University of Auckland, Auckland 1023, New Zealand; 3 Huntly West Pharmacy, Hamilton 3700, New Zealand; samuel.martin1707@gmail.com; 4 Waitematā Hospital, Auckland 0610, New Zealand; Owen.Sinclair@waitematadhb.govt.nz; 5 Department of General Practice and Primary Health Care, University of Auckland, Auckland 1023, New Zealand; h.petousis-harris@auckland.ac.nz; 6 Waikato District Health Board, Hamilton 3204, New Zealand; Felicity.Dumble@waikatodhb.health.nz; 7 General Paediatrics, Starship Children’s Hospital, Auckland 1023, New Zealand

**Keywords:** maternal vaccination, health care professional, midwifery, general practice, primary care, community pharmacy services, access to medicines, pertussis, influenza, health policy, pregnancy

## Abstract

The uptake of maternal influenza and pertussis vaccinations is often suboptimal. This study explores the factors influencing pregnant women’s and health care professionals’ (HCPs) behaviour regarding maternal vaccinations (MVs). Pregnant/recently pregnant women, midwives, pharmacists and general practice staff in Waikato, New Zealand, were interviewed. The analysis used the behaviour change wheel model. Interviews of 18 women and 35 HCPs revealed knowledge about MVs varied with knowledge deficiencies hindering the uptake, particularly for influenza vaccination. HCPs, especially midwives, were key in raising women’s awareness of MVs. Experience with vaccinating, hospital work (for midwives) and training increased HCPs’ knowledge and proactivity about MVs. A “*woman’s choice*” philosophy saw midwives typically encouraging women to seek information and make their own decision. Women’s decisions were generally based on knowledge, beliefs, HCPs’ emphasis and their perceived risk, with little apparent influence from friends, family, or online or promotional material. General practice’s concentration on children’s vaccination and minimal antenatal contact limited proactivity with MVs. Busyness and prioritisation appeared to affect HCPs’ proactivity. Multi-pronged interventions targeting HCPs and pregnant women and increasing MV access are needed. All HCPs seeing pregnant women should be well-informed about MVs, including how to identify and address women’s questions or concerns about MVs to optimise uptake.

## 1. Introduction

Pertussis and influenza vaccinations during pregnancy are recommended and funded in many countries [1,2]. Pertussis (whooping cough) causes hospitalisations and deaths, particularly of young infants [3,4]. A tetanus–diphtheria–acellular–pertussis (Tdap) vaccination during pregnancy protects young infants against severe pertussis [5,6].

Increased influenza-associated mortality and hospitalisations [7] and adverse fetal outcomes are associated with influenza during pregnancy [8]. Maternal influenza vaccination is associated with a reduced risk of influenza-associated hospitalisations of pregnant women [9] and infants [10].

The uptake of maternal vaccinations (MVs) is suboptimal in many countries [11,12,13,14], particularly among those experiencing greater socioeconomic deprivation [15,16] and among indigenous people and people born overseas [16,17]. Lack of awareness, misperceptions, safety concerns and access challenges hinder maternal vaccine uptake [14,18,19,20,21,22,23,24,25]. Conversely, promotion and administration of MVs by antenatal care providers can increase the uptake [26].

While health care professional (HCP) recommendation and/or endorsement of safety encourages uptake [12,18,19,23,27], sometimes antenatal care providers do not inform people about MVs [12,13,19], with some providers lacking confidence and/or knowledge [21,22,28,29,30]. Specific training about MVs to health professionals increases their confidence to give appropriate advice to pregnant women [31,32].

A Canadian survey found that recommendations for influenza vaccination made to pregnant women varied among HCP groups, with midwives (38%) less likely than pharmacists (70%), nurses (84%) or physicians (80%) to report that they recommended the influenza vaccine to all their pregnant patients [33]. In Ireland, pregnant women reported recommendation by general practitioners for maternal pertussis vaccination occurred more frequently than recommendation by obstetricians, midwives, or nurses [23].

In New Zealand (NZ), maternal influenza vaccination uptake in 2018 was 31% and maternal pertussis vaccination uptake was 44% [16]. While steadily increasing, the uptake remains suboptimal, and is particularly low for Māori (indigenous people in NZ) and Pacific peoples, those living in more deprived circumstances and for younger women. However, NZ research that investigates the reasons for this low vaccine uptake has been limited to maternal pertussis vaccinations, and has to date considered views sought from pregnant women, but not from HCPs [19,34]. Conducted soon after maternal pertussis vaccinations became government-funded, this research shows that women had low awareness of the vaccine [19,34]. Quantitative research in NZ completed as part of the project reported in this manuscript found that extending funding to pharmacies increased the uptake of maternal pertussis vaccinations, indicating improving access could help [35]. Vaccine uptake remained low, however [35]. Thus, there is a need to understand the factors in NZ that influence HCPs’ discussions with pregnant women about MVs and that influence women’s MV uptake.

International research published to date has focused on either pregnant women alone [12,13,23,24], or a single HCP group [28,30,36,37,38], or multiple HCP groups [20,31,32,33]. Infrequently, data from women and HCPs have been reported from the same study. Wilson et al. [39] interviewed 40 women and 10 HCPs in Hackney, London, to identify strategies to address low MV uptake. The resulting recommendations included reducing appointment wait times, providing more in-depth personalised vaccination discussions, encouraging friends and family to be informed about MVs, and better midwife continuity for the patient. The generalisability of the findings was limited by short interviews with a small number of HCPs and the location in a single London borough. Wilcox et al. [21,22] surveyed antenatal care providers and pregnant women about MVs uptake in South England, examining opinions on vaccine protection, provider confidence and women’s reasons for non-vaccination. The education of HCPs was considered essential, increasing their confidence in discussing MVs. Of note, despite logistical challenges found with provision of vaccines by primary care, there was little support for provision elsewhere [21].

In order to increase MV uptake, there is a need for countries, including NZ, to conduct in-depth studies of perspectives, experiences and behaviours of women in late pregnancy or who have recently been pregnant, and of HCPs providing antenatal care or who administer MVs. Such studies would increase the understanding of the factors determining MV uptake and HCP discussions regarding MV and inform interventions that seek to increase MV uptake.

The Capability Opportunity Motivation-Behaviour (COM-B) framework within the behaviour change wheel model [40] has been used to analyse health-related behaviours, including barriers and enablers to vaccine uptake [41,42,43]. This framework has three essential conditions for behaviour: capability (the psychological and physical capacity for a behaviour, including necessary knowledge and skills); opportunity (factors outside the individual that make behaviour possible or prompt it, such as physical opportunity and social opportunity); and motivation (brain processes that energise and direct behaviour, including automatic processes as well as reflective processes involving evaluation and plans).

In this manuscript, barriers and enablers for MV uptake are explored in NZ, using the COM-B model, through interviews with pregnant or recently pregnant women and the HCPs that most commonly provide or recommend MVs.

## 2. Materials and Methods

### 2.1. Study Setting—New Zealand and Specifically the Waikato Region

New Zealand is a developed country with an estimated population of 5.1 million [44] of which 70% identify as European, 17% as Māori, 15% as Asian and 8% as Pacific peoples [45]. New Zealand has universal health care that includes fully funded hospital care, primary maternity care, and vaccinations (i.e., no cost to the patient). Prescriptions and general practice visits are partially funded, with a patient co-payment. Most of the population is enrolled with one identified general practice. Pregnant women can choose a Lead Maternity Carer (LMC) to provide maternity care from early pregnancy through to labour and birth and for up to six weeks post-partum. Most maternal care, including pregnancy, birth care and care of the infant up to age six weeks are provided by midwives (94% of women’s LMCs). The use of a private maternity specialist (6% of women’s LMCs) incurs a patient charge. Most LMC midwives work independently of the hospital, typically in small practice teams of 2–6 midwives. Midwives (core/hospital) also work within hospital settings, in this role providing midwifery support for the LMC along with care of women who have complex pregnancies as members of the hospital maternity team. Typically, a single midwife provides the health care for a woman throughout her whole pregnancy.

The Waikato District Health Board (DHB), a central North Island region of NZ, serves a population of over 426,000 people (about 9% of NZ’s population) [46], 58% of whom live in urban areas [47]. Of the approximately 5400 women who give birth annually, 37% identify as Māori and 34% are in the most deprived quintile of NZ. There is one city, Hamilton, with a population of 170,000, and 3 towns with populations from 10,000 to 20,000.

In NZ, specified vaccinations are government funded, including their administration, through general practice, and free for the patient. Two MVs have been government funded through general practice and hospitals nationwide since 2010 (influenza) [48] and 2013 (pertussis) [49]. In late 2016, maternal pertussis vaccinations became funded in pharmacies in the Waikato DHB region only. In 2017, maternal influenza vaccinations became funded nationally through pharmacies. In general practice, an appointment is usually required for a vaccination with the vaccine given by a practice nurse. Appointments are usually only available Monday to Friday during business hours.

In 2017, about a quarter of all NZ community pharmacists were qualified to provide vaccinations [50]. Pharmacies providing vaccinations usually do not require an appointment if a vaccinating pharmacist and first-aid qualified staff member are on-site. All pharmacies are open Monday to Friday, and many are open at least on Saturday mornings, with some open seven days per week, sometimes including evenings. In the Waikato DHB region in 2018, 35 of the 83 community pharmacies provided vaccinations, including a larger proportion of those in urban areas (60% of pharmacies in urban areas versus 21% in rural areas; data from this current study).

LMC midwives do not usually administer MVs in their clinics. Nurses, pharmacists and midwives who provide vaccinations undergo the same vaccination micro-credentialling with a mandatory refresher every two years and must meet the Immunization Standards for Vaccinators.

### 2.2. Study Design

This paper reports on part of a mixed methods study that aims to evaluate the provision of MVs in pharmacies and understand the barriers and enablers to MV uptake. The qualitative research published to date from this study has described the perspectives and experiences of women and HCPs with respect to funded MVs in pharmacies [51]. The quantitative results are published elsewhere, and showed significant gaps and inequalities in MV uptake [35].

Semi-structured interviews were conducted in the Waikato DHB region from November 2018 to May 2019. The participants came from four categories: women who were at least 20 weeks’ gestation or recently pregnant; midwives; general practice staff; and community pharmacists.

### 2.3. Recruitment and Interview Process

Participants were purposively selected to capture a wide range of experiences and perspectives, including rural and urban locations, and various ethnicities. Rural areas and high-deprivation areas, and people of Māori ethnicity were deliberately over-sampled given their potentially greater challenges of health care access.

Most women were recruited through pharmacists, with one recruited through a midwife. Diversity in age, geographical location, MV status, place of vaccination, and number of previous pregnancies was sought. All participants needed sufficient English to be interviewed.

Health care providers were selected for variation in their experience and geographical location. We sought Māori health care providers, owner and non-owner GPs and pharmacists, and independent midwives and midwives currently or previously working in public hospitals. The College of Midwives regional coordinator helped the researcher to identify a range of midwives to invite to participate.

As practice nurses administer most vaccinations in general practice, more nurses (all vaccinators) were invited into the study than GPs. Pharmacists who administered vaccinations were approached for most interviews and two rural pharmacy owners not offering vaccinations were also invited to participate.

Following informed consent, interviews were conducted face to face at the participant’s workplace, in their home, in a pharmacy consultation room, or at a café, or by telephone according to the participant’s preference and availability. A NZD 30 voucher was provided to each participant. The topics of discussion for women included: knowledge about pertussis in babies, influenza in pregnancy, and MVs; sources of information about MVs; opinion on vaccines in pregnancy; whether MVs were received during their latest pregnancy and why/why not; the patient journey and experience of receiving the vaccination, and any challenges to/concerns about receiving the vaccine/s; views on vaccination generally; and demographics. The topics of discussion for HCPs included: their role in vaccination; experience administering vaccinations (if applicable); knowledge about MVs; barriers and enablers to women receiving MVs; how to increase MV uptake; barriers and enablers to provide or recommend MVs; and demographics. See Supplementary Materials File S1 for the questionnaire guide questions relevant to this paper. Interviews were audio recorded (with consent) and then transcribed and checked against the recording. Where audio recording was not permitted, notes were taken.

### 2.4. Analysis

Transcripts and notes were read and reread by the first author, then coded using NVIVO Pro. The interviews were coded within four groups: women consumers; midwives; pharmacists; and general practice staff. Within each group, coding nodes included specific topics discussed (deduction) and emerging themes (induction). For example, under the coding of women consumers were codes such as barriers to receiving the vaccine, enablers to receiving the vaccine, knowledge, and the woman’s journey, and themes such as protect baby, empowerment, safety, and trust. All data were coded into one or more nodes. The analyses involved systematically working through each coding node for each of the four groups. The emerging themes were documented. The findings were then grouped according to the COM-B model, moving back and forth between the data (in the coding nodes and interviews) and the understandings to make optimal use of the evidence contained in the data [52]. Comparisons were made between and within groups, looking for similarities and deviant cases, and providing a level of triangulation for comparisons between the four study participant groups.

We did not aim for data saturation, but rather for a breadth of findings from a range of different participants representing different groups.

### 2.5. Researchers’ Roles and Perspectives

Following training, the second author, a male Māori pharmacist, interviewed the Māori and Cook Island Māori women using the interview guide, receiving feedback following the first interviews. The first author, a female NZ European pharmacist and experienced interviewer, conducted all other interviews. The first author coded the transcripts, conducted analyses and reported findings, with input from the second author and a third author (OS) on the Māori women findings.

Both interviewers tried not to reveal their stance on vaccination to participants, rather seeking to understand and encourage participants to share their views. While pharmacists were aware that their interviewer was also a pharmacist, other health care providers and most women were not. A minority of the Māori women had an existing relationship with the interviewer as their pharmacist.

All authors had input into the study and reviewed the findings before being finalised. HPH is a female NZ European vaccination researcher, CCG is a male NZ European academic general paediatrician, OS is a male Māori general paediatrician practising in hospital, AH is a data analyst and researcher, and FD is a public health physician. All authors hold positive views about vaccination.

## 3. Results

### 3.1. The Sample

A total of 18 women participants, 12 pharmacists, 12 people working in general practice, and 11 midwives were interviewed (totalling 53 interviews). Nine of the women participants and nine of the health care providers were Māori. One woman had her partner with her during the interview, with this partner making some comments. No other participants had adults with them at the time of the interview.

The characteristics of the interviewed women are summarised in Table 1, and the characteristics of the interviewed health care providers in Table 2. Māori women who were interviewed tended to be younger and have more children than the other women. Five of the eighteen women reported receiving both pertussis and influenza vaccinations in pregnancy, one planned to receive both, eight reported receiving the pertussis vaccination only, and one reported receiving influenza vaccination only. Three women received no MVs.

All 12 pharmacists approached agreed to participate. Of the 12 midwives first approached, 10 agreed to be interviewed, one was no longer practising, and the other was difficult to contact. One further midwife was recruited through snow balling. All seven general practices approached provided a practice nurse and/or GP; one provided a practice manager and practice nurse.

Forty-seven interviews were conducted face to face, and six by telephone. Most interviews took around 25–30 min (range 7–52 min). Two participants declined audio recording with notes taken instead.

The coding of participants indicates their subset with W for woman (W1-W18), M for midwife (M1-M11), P for pharmacist (P1-P12), N for practice nurse (N1-N7), PM for practice manager, and GP for general practitioner.

The results are presented using the COM-B model behaviour framework (capability, opportunity, and motivation), divided into the different participant groups interviewed.

### 3.2. Capability Opportunity Motivation-Behaviour (COM-B)

**Figure 1:**
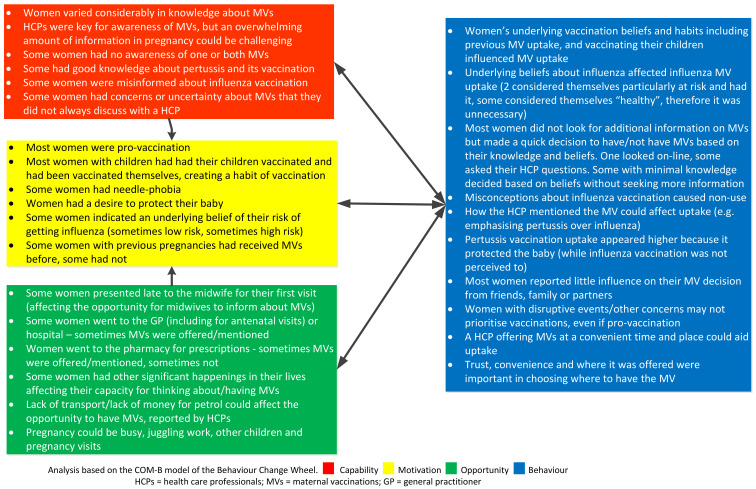
Factors influencing the uptake of maternal vaccines (MVs) for women using the COM-B model.

**Figure 2:**
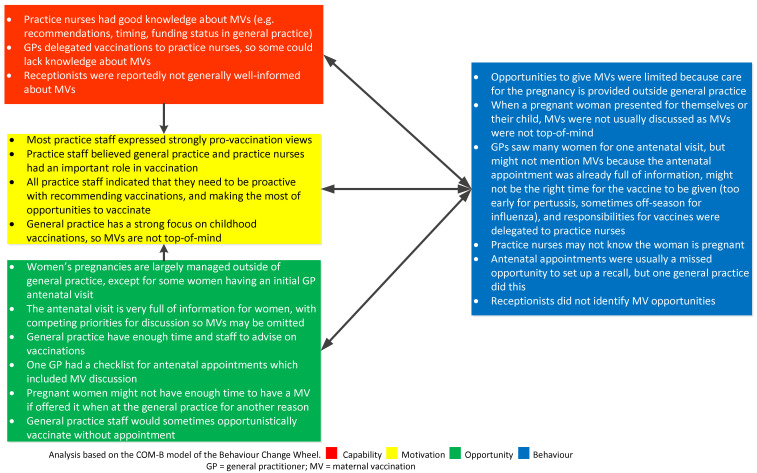
Factors affecting the recommendation and administration of maternal vaccines (MVs) for general practice staff using the COM-B model.

**Figure 3:**
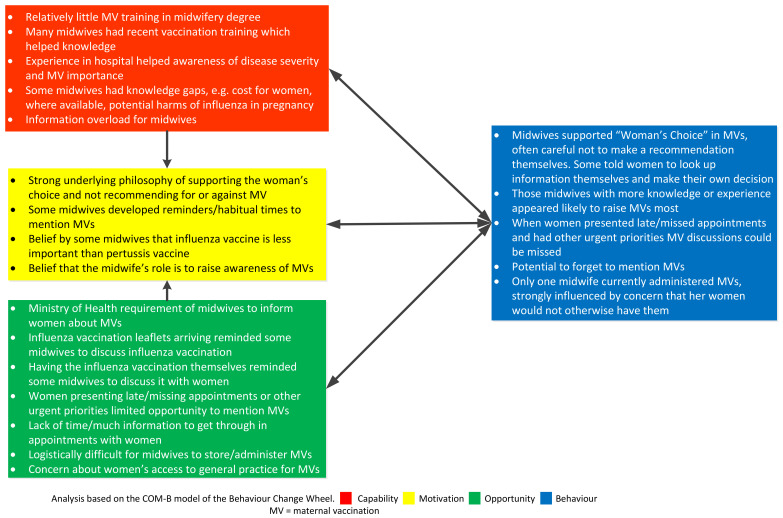
Factors affecting the recommendation and administration of maternal vaccines (MVs) for midwives using the COM-B model.

**Figure 4:**
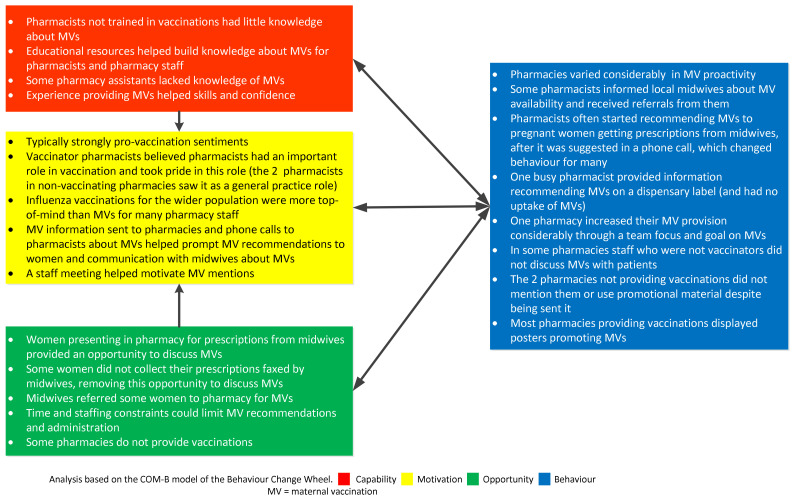
Factors affecting the recommendation and administration of maternal vaccines (MVs) for pharmacists using the COM-B model.

Using the behavioural change wheel COMB-B model, comparisons were established between the four groups interviewed in terms of common or salient influences on behaviour regarding having MVs or recommending MVs and are summarised in Figure 1, Figure 2, Figure 3 and Figure 4.

#### 3.2.1. Women’s Behaviour with Respect to Maternal Vaccinations

Fifteen women reported having had one or both MVs (or intending to) and three women had no MVs. Five women reported having both MVs, and one was intending to (22 weeks’ gestation at time of interview); eight only had a pertussis vaccine, and one woman only had an influenza vaccination.

Women typically described a seemingly straight-forward decision regarding MVs. Most were informed by a HCP, sought no other information on MVs, and their decision as to whether to have a MV appeared to be primarily based on underlying beliefs, previous vaccination activity (habits), and their knowledge. Lack of awareness appeared to be the most common barrier to receiving pertussis vaccination.

Reasons women gave for not having the influenza vaccination differed from those for the pertussis vaccination, appearing to be related to misconceptions (underlying beliefs), seasonality (availability and HCP information), and less emphasis by HCPs than for the pertussis vaccine.

Two women and some midwives spoke about the challenges of other occurrences in the lives of some pregnant women that affected their ability to prioritise MVs, or even to learn about them. This is discussed further in the Opportunity Section, below.

Indicating the multi-factorial nature of MV uptake, one NZ European woman received no MVs despite awareness of MVs and describing herself as pro-vaccination. For this woman, other concerns were top-of-mind making MVs a low priority. These concerns included work, housing challenges, other medical conditions, and child responsibilities. She was unaware of the benefits of the influenza vaccination in pregnancy, thought she was not at risk, and was reluctant to have “*another needle*”. She saw MV posters and read a leaflet on influenza vaccination. Although some HCPs she saw did not mention MVs, at least two HCPs mentioned MVs, but they did not offer to administer them at the time of discussion. She noted that, had she been offered the vaccines on the spot at the hospital or the pharmacy or had someone offered to vaccinate her at her home, she would have had them. It needed to be easy for her.

*“If I could have just gotten it done [at the pharmacy or hospital] all then and there it would have been fine. You don’t have to think about it, you don’t have to go anywhere. It’s done.”*

W10

This example highlights that simply raising awareness may be insufficient, and dialogue about MVs, identifying and addressing concerns, and offering the MVs while the woman is already waiting, e.g., for a prescription or for an appointment at the hospital, could increase the understanding of the benefits and aid the uptake.

#### 3.2.2. Capability for Women

Women’s awareness and knowledge emerged as vitally important in enabling them to have MVs. Women who did not have one or both vaccinations usually had knowledge deficiencies regarding MVs, including lack of awareness of the recommendation to have the vaccine or misconceptions about the vaccine or the potential harm from the disease (explained further below).

HCPs were the key source of awareness and knowledge for those who received vaccinations. Midwives were the HCP most likely to inform women of MVs and often first mentioned by women as a source of information. As the primary information source, it is likely that the capability of HCPs could affect the capability of pregnant women.

Some women reported receiving useful information from the midwife about when to have the MV/s, that the MV protects the baby, and where to receive MVs. One reported that all her MV questions were answered by the midwife, instilling confidence to have MVs. A few women reported multiple conversations with the midwife, e.g., a mention early in the pregnancy and a later reminder to receive it as the administration time arose later in the pregnancy (for pertussis). Some women indicated the midwife was quite influential.

*“…all through my pregnancy of course my midwife recommended… all the shots…”*

W8 (influenza vaccine only)

In contrast, two women participants suggested their midwife could be better informed, and five women reported receiving no information from their midwife. However, they wanted midwives to tell them about MVs.

General practice and pharmacy also appeared to be important, including being the sole source of MV information for several women. However, some women did not attend the general practice during pregnancy, and some reported no MV discussion when attending general practice or pharmacy.

*“I feel like I’ve had to seek all the information out… I think my first midwife was maybe a bit anti-vaccinations, I got that vibe from her… I think I got a hand-out about whooping cough. And then the second time round… my midwife hasn’t mentioned it at all I don’t think. Then I went to the pharmacy for something else, and the pharmacist just popped her head over and she’s like oh are you pregnant?... [R]eminded me that I needed to get the whooping cough the second time round, coz I actually didn’t know...”*

W11 (pertussis vaccine only)

Traditional media, social media, leaflets, posters, friends, and family were mentioned as sometimes providing information, but were supplementary to the HCP informing the patient and never emerged as a significant source of awareness or information about MVs. Some women had multiple sources of information, e.g., a NZ European woman reported the midwife, pamphlets, birth centre, other mothers, and family raised awareness of pertussis. Online information, including social media, appeared to have minimal influence in raising awareness or providing information with several respondents not trusting it, and only one woman reported looking up information about MVs on-line.

*“I don’t think I trust much on Facebook myself. I’d be more inclined to probably book an appointment with the doctor or something and ask all those questions.”*

W1 (neither MV)

A midwife thought women would have difficulty finding balanced information online on MVs, given the challenges she had found herself.

Several women participants who presented late to the midwife lacked awareness of pertussis vaccination and midwives reported vaccination information might be more limited or overtaken by other priorities in such circumstances. Some HCPs reported some women had no awareness when the HCP mentioned MVs. Some midwives and a woman participant worried that messages about MVs would be lost given that the amount of information provided in antenatal visits could be overwhelming:

*“…we can only do so much because immunisations [are] only a small part of our discussions and a lot of women aren’t focused on immunisations when they’re so scared of the… birth.”*

M2

Knowing that the vaccination protected the baby encouraged women to receive the pertussis vaccine, but the influenza vaccination was often not perceived to help the baby, and often considered less important.

Misconceptions commonly contributed to decisions not to have an influenza vaccination. These included: thinking: they were healthy and therefore not at risk if they had an influenza infection (unaware of pregnancy as a risk factor); that the influenza vaccination causes influenza infection; or that the influenza vaccination is ineffective. One woman reported a pharmacist said it was unnecessary, being early summer (and did not have it). Another who had the pertussis vaccine did not want an influenza vaccine because she did not know enough about it, but sought no information. A further self-described pro-vaccination woman had the pertussis vaccination (informed by another woman and her midwife), but not influenza:

*“… if… my midwife or someone told me I need to be vaccinated against influenza then I would have done it.”*

W18

One midwife reported that language difficulties for some Indian, Somali, or Cambodian clients impacted their ability to understand vaccination information.

#### 3.2.3. Capability for Health Care Professionals

Many of the HCPs knew both pertussis and influenza vaccines were recommended in pregnancy, knew they were free from general practice, and knew the recommended timing of the pertussis vaccine, and discussed their benefits, particularly where they had experience and training in providing adult vaccinations or MVs specifically. Many HCPs could administer vaccinations, although the GPs delegated this task to nurses, and two pharmacists did not administer vaccinations. Only one midwife was currently administering vaccinations.

Practice nurses were generally well informed on MVs, although one wanted more information, and most did not know maternal pertussis vaccinations were funded through the pharmacy. One GP reported that her recent experience in obstetrics and gynaecology during training provided excellent knowledge about MVs, but the other GPs had concerns about their lack of knowledge, primarily because vaccination is delegated to nurses:

*“I forget about these vaccines always. And I go to nurses and they know, and I can’t hold all, so much information…”*

D2

Some midwives were well informed on MVs, e.g., the recommendation to have both MVs in pregnancy, timing, that they were free, and their benefits. However, some noted that their midwifery training on vaccination was relatively minimal. A couple were unsure whether MVs were free for women in general practice, and one wanted more knowledge about maternal influenza vaccination. While some midwives had seen bad cases of influenza in pregnancy or pertussis in infants, which helped them to understand the risks of not having the vaccination, others had not.

*“… the biggest barrier for a lot of midwives is that you don’t know and… you don’t want to talk about things when you don’t know…”*

M3

*“… even midwives don’t understand [how bad influenza in pregnancy can be] because we don’t see it.”*

M11

Some midwives noted some knowledge gaps, including the benefits of maternal influenza vaccination. Many recommended women find more information for themselves.

*“[Women] have this misconception of when I get the flu vaccine I get the flu... I don’t really know how to kind of demystify that…”*

M2

One woman reported her midwife had not told her about MVs:

*“I told my midwife [I’d had the vaccination]… she had heard about it but doesn’t actually know much about it…”*

W4 (pertussis only)

Some midwives reported being busy and having information overload hindered learning more about MVs, but most midwives sought further training since graduating, gaining knowledge and confidence with MVs.

One midwife noted students and graduates were influenced by the last midwife with whom they worked, who may be less positive about vaccinations. Another midwife was concerned about the lack of long-term data with MVs.

The two pharmacists who were not providing vaccinations in their pharmacies lacked knowledge about MVs and did not raise MVs with patients. The vaccinator-pharmacists recalled the pertussis vaccination timing well, *“we have to repeat it…a lot…”*. Some pharmacists ensured non-pharmacist staff were informed about MVs, e.g., with staff meetings, having MV key points or posters displayed on the wall for staff, or targets, with some staff members recommending to clients to have MVs.

#### 3.2.4. Opportunity for Women and for Health Professionals

Women’s access to MVs was aided by the opportunity for women to be vaccinated in the hospital and in pharmacy. However, logistical challenges prevented most midwives administering MVs, e.g., maintaining a cold chain, being busy, not receiving additional funding for vaccination administration, and knowing an urgent call out was incompatible with the 20 min post-vaccination observation. While some midwives provided services from a birthing centre, and one participant suggested MVs could be provided from such a facility, one midwife was negative about the idea, thinking it would be too *“pro-vaccination”,* affecting the woman’s choice to have MVs. Two rural pharmacies where the non-vaccinating pharmacists worked did not provide a vaccination service, so did not aid access.

One hospital midwife had started to provide MVs on concern about challenges for women in being able to attend the doctor, reporting that 80–85% of her women now receive MVs.

*“… because we’ve got the vaccines on site it’s just so much easier and the women go ‘yup, let’s do it.’ I do it at the beginning of our appointment so by the time we’ve finished… they’ve had their observations and they like it like that.”*

M1

Some women and midwives interviewed believed that requiring a general practice appointment significantly hindered MV uptake, given the busyness of pregnancy, working full time, having other children to manage, other distractions such as health issues, and lack of telephone reception or funds on a mobile phone.

*“… more pressing issues like, I don’t know, the grocery shopping or getting all my five kids ready for school and you know it’s things that are like more in the forefront of their mind rather than this vaccination that they should probably think about getting.”*

M3

*“I complain sometimes about my ladies not coming here but sometimes the pregnancy is the smallest issue in their life… Some of it is drugs and alcohol and priorities being askew.”*

M5

General practice participants reported that their practice would provide vaccinations without appointments when an opportunity to deliver vaccinations arose, reducing barriers to those present in the general practice, but with women and midwives unaware of this facility, this barrier remained.

When pregnant women went to the general practice, pharmacies, or hospital, some reported that they were not offered a vaccination on the spot, nor (sometimes) were they informed about them. General practice participants said unawareness of a woman’s pregnancy limited their opportunity to provide MV advice or administration. However, some general practice participants observed that many women have an early antenatal visit in the general practice and the practice could implement a text reminder for a MV; only one practice nurse did this though. MVs did not appear to be administered at the first antenatal visit generally.

Some midwives noted travelling to receive the vaccine could be difficult, particularly if living rurally, without a car, and/or with little money.

Non-vaccinating pharmacy staff members or general practice receptionists who were unaware of MVs could not mention them to women.

For the influenza vaccination, provision and recommendation was limited by the season with none usually available from approximately December to March. The pertussis vaccination only was funded from 28 to 38 weeks’ gestation during the period of the interviews. Some participants suggested that a longer window of availability might help this vaccine to be given opportunistically.

#### 3.2.5. Motivation for Women and for Health Professionals

The motivation for women to have MVs reflected underlying beliefs (e.g., pro-vaccination, beliefs about the severity of influenza), habit (vaccination in the last pregnancy), emotional responses (e.g., being scared about the effect on the baby, needle phobia), and conscious decision making (e.g., considering their personal level of risk).

Motivation was affected by capability and opportunity. For example, insufficient knowledge, including lack of awareness about MV/s, potential harm from influenza or pertussis, or understanding of the benefits of the vaccine could affect the woman’s motivation to have a MV or the HCPs proactivity with one or both MVs.

Lack of time and other distractions sometimes impacted motivation and therefore uptake for women and motivation and proactivity with MVs for HCPs. However, there were cases in which midwives made time to be proactive with information even when busy, where they saw it as important. One midwife became motivated to administer MVs in her hospital role, concerned that women would not otherwise receive them in part because of the challenges for women to obtain an appointment for MV administration at the general practice.

*“… at first [on administering MVs] it was like I did sort of hesitate a little bit… It’s actually knowing that these women are being vaccinated on time and helping to protect the babies, that’s made me realise now that it’s actually an important part of the role.”*

M1

Typically, a midwife and/or other HCPs told women about one or both MVs. Those women then appeared to seek no further information from any other sources—the internet, reading materials, or asking their partner, friends or family. A few women reported asking the HCP questions. For most women, this appeared to be a simple decision with little angst, with only a couple of deviations from this. Women could not be motivated to have the vaccination/s if unaware of them.

A small number of women participants believed that MVs were unnecessary.

*“I still feel that females were made to do it [carry a baby] I suppose.”*

W1 (received neither MV)

One of the doctor participants reported some clients hesitated about MV (but not other vaccinations) with an apparent *“feeling of over-medicalising the pregnancy”.* Several other health professionals also indicated some women appeared to need reassurance:

*“I’ve turned so many around when I’ve said, “oh actually it’s recommended” and I show them the posters… and they go ‘oh okay that’s fine because my midwife has suggested it but I’m still a bit…”*

P10

Most women indicated they had pro-vaccination perspectives making them favour MVs in most cases (with one exception mentioned earlier affected by other concerns).

*“I quite like being vaccinated, especially during pregnancy and for baby after baby’s born. I find it most important that my babies are vaccinated when they should be.”*

W12 (planning to have both MVs)

The perceptions of others’ experiences could affect their motivation, for example awareness of children with medical issues during infancy made two women particularly keen to receive MVs to protect their babies. Women with previous pregnancies without MVs appeared to have low motivation to have them this time, possibly reflecting that a habit had been formed.

Perspectives could change during the pregnancy. W8 was told about both vaccines by her midwife, and reported the midwife was the primary reason why she had the influenza vaccination with little consideration as winter approached. The midwife suggested pertussis again, noting a nearby pertussis outbreak, but the woman declined (after research and discussion with her partner) because she had had two health scares during her pregnancy (one a week after the influenza vaccination) and worried the pertussis vaccine could cause harm. *“So, I guess I was kind of like I’ve got enough to deal with as it is, I don’t want to have another scare with a shot”*. However, her baby received its recommended vaccinations, albeit deliberately delayed.

Reminders helped to motivate women to receive the vaccinations, most commonly HCP verbal messaging when the pertussis vaccine was due. Posters in the pharmacy were mentioned by a couple of participants and one participant reported receiving text reminders.

*“I got little text reminders on my phone from the doctors to remind me when my appointment was due to go and get them [MVs] done too, [handy] [e]specially when you’ve got so many children and after one you start losing track.”*

W12 (planning to have both MVs)

Women usually reported little or no influence from media and other people—partners, friends or family. Some women stated that it was their body and therefore their decision and did not discuss MVs with their partner or others. Even a teenage participant having her first baby sought no advice on MVs from her parents and partner, despite living with them. Some women described being uninfluenced by opposing views from family or friends. One woman had no MVs despite a pro-vaccination mother, another had MVs despite a vaccination hesitant mother, and a third had a MV despite having vaccine-hesitant friends. However, one woman was prompted to have her pertussis vaccine after members of her antenatal group mentioned having it.

Where HCPs did not recommend the influenza vaccination or were perceived to be a little less convincing about it, sometimes women perceived these vaccinations were unimportant. Participant W15 received the pertussis, but not the influenza vaccine. Although the pharmacy mentioned both vaccinations, the influenza vaccination was not given because *“the hospital never said to get it done”* and the pharmacist suggested it was unnecessary being summer. Another woman had the pertussis vaccination on her midwife’s advice, but not the influenza vaccination: *“it was sort of if you want to you can, but…”*. One midwife reported telling her women: “*if you get this [influenza] during pregnancy you are much more likely to end up in hospital*”, but most women did not seem to understand the benefits of influenza vaccination specific to pregnancy.

An important aspect permeating through most interviews with midwives was a strong underlying philosophy that the woman’s decision about MVs was *“her choice”* and the midwife’s role was not to make a personal recommendation about what to do, but to support her to make her own choice. Some midwives recommended their women *“go have a look [for information] for yourself”.* Others wanted women to have plenty of time to consider MVs. There was often a desire expressed not to be “*pushy*”.

*“I usually say Ministry of Health recommends because I don’t want it to be like I’m pushing this on you because I’m not, it’s up to you to make that choice.”*

M3

*“… we’re really passionate about giving choices and options so on some topics that’s really hard… you want to give them the information, but you can’t be too directive.”*

M6

When vaccinations were raised with midwives in interviews, the first comments were always about childhood vaccinations, reflecting that these were the strongest focus for them, although most also noted their contractual requirement from the Ministry of Health to inform about MVs.

Unlike midwives, nurses and pharmacists providing vaccinations expressed no concerns about women’s choice except two pharmacists who gave women choice about where to get their vaccination: *“… we’d rather they get it done anywhere… than not getting it done at all”*.

General practitioners saw vaccination as an important role for the general practice, but largely delegated it to nurses. General practice participants described how the general practice prioritised vaccinations and would try to give on the spot if an opportunity arose (without requiring an appointment). Childhood vaccinations being the key focus (partly because of targets), and ongoing pregnancy care being the focus of midwives rather than the general practice, MVs were not top-of-mind for general practice. General practice participants said sometimes the first time they knew of the pregnancy was when the woman attended for the baby’s six-week check-up. Time pressure did not help. One general practitioner called MVs *“one of the less important of my tasks to do”*.

*“… the chance is we might forget about the immunisation because there’s a lot to discuss on the [antenatal appointment] with the excitement and we have 15 min.”*

D2

However, one general practitioner’s experience in obstetrics and gynaecology heightened her motivation with MVs. She always discussed MVs with women in their antenatal visits, aided by a checklist.

Proactivity by pharmacists and other pharmacy staff in discussing MVs (e.g., when seeing antenatal folic acid or iodine prescriptions) reportedly improved the uptake. One very busy pharmacist provided brief written information rather than discussing MVs, with no resulting MV uptake, possibly reflecting the importance of verbal communication.

Experience in administering vaccinations in adults, recent education on vaccinations, and those regularly administering MVs engendered confidence with discussing vaccinations. Two midwives gained confidence from administering MVs while working in hospital:

*“… when you start giving 14 vaccines a day you… get confidence in it and you begin to realise that it is safe.”*

M4

## 4. Discussion

### 4.1. Summary of Findings

This study identified barriers and enablers to MVs during pregnancy through the exploration of the woman’s perspectives alongside HCPs’.

In this study, HCPs (particularly midwives) emerged as the most important influence on the uptake of MVs in women with little apparent effect from promotional material or friends or family. HCPs raising awareness, making recommendations, providing information, answering questions, and offering MVs at appropriate times all appeared to enable the uptake of MVs. Multiple HCPs played an important role, reinforcing messages or in the cases when women had not remembered receiving MV messaging from other HCPs. Women were often sensitive to a HCP’s messages and how they were delivered. Simply raising awareness without discussion about benefits and risks did not convince some women to have a MV. Similarly, delivering a message that appeared to a pregnant woman to be ambivalent about a MV sometimes appeared to discourage the uptake.

Women’s choice was an important theme that emerged strongly from midwives and women. For women, “*my body, my decision*” made the decision on MVs theirs alone, typically without conferring with family or friends, and often uninfluenced by others’ vaccination perspectives. Midwives had a strong philosophy of women’s choice, such that most were very careful not to lead the woman in any way in their MV decision, but often encouraged them to find out more information for themselves. Some women thought a midwife had not favoured a MV or been ambivalent, possibly because of how the messaging was provided.

Important differences emerged between women and HCPs about pertussis and influenza vaccinations. HCPs often appeared to emphasise the pertussis vaccination more than the influenza vaccination. Most women perceived they were healthy and therefore not at risk of influenza, and no woman was aware of the higher risk of hospitalisation with influenza during pregnancy (compared to non-pregnant women), with few HCPs communicating this risk to their women. The influenza vaccination was perceived by many women to be for the woman rather than for the baby, while the pertussis vaccination was known to protect the baby and therefore prioritised by some women. Influenza vaccination seasonality and lack of availability in summer also affected the uptake.

HCPs often described the challenges to finding enough time or remembering to discuss MVs, particularly in light of the amount of overwhelming information in general practice ante-natal appointments and midwives’ appointments, busyness of other work, and sometimes late first presentation of women to the midwife and more urgent needs for women. Furthermore, general practices prioritised childhood vaccinations, with MVs far less considered. Women and HCPs sometimes noted challenges for women from the busyness of pregnancy, juggling work and/or other children, and appointments. Recalls for MVs from the general practice, availability without appointments and outside of regular work hours, and HCPs offering MVs opportunistically could help to overcome this. Sufficient information and a strong underlying pro-vaccination philosophy helped to make MVs a greater priority for women and HCPs. HCPs with more knowledge and experience relevant to MVs tended to prioritise these conversations more.

There were many factors with MV uptake that were woven together to influence women’s behaviour, including beliefs, knowledge, experience, and opportunity to receive the vaccine, which affected their decision/s about and their prioritisation of MVs. Similarly, for HCPs various factors influenced their messaging about MVs. Knowledge and experience were particularly important and those with more training or experience prioritised MV discussions and had good confidence about these discussions.

### 4.2. Comparison with Other Literature and Implications

Research in NZ [19,34] and elsewhere [20,53] shows that lack of awareness hindered the uptake of the maternal pertussis vaccination. While some women in this study remained unaware of one or both MVs, particularly those who presented late in their pregnancies for care, we found many women were informed about MVs by at least one and often multiple HCPs. This is consistent with the increasing uptake of MVs seen [16] since the earlier NZ research.

Others have also found misconceptions about the vaccine or risks of the disease, or insufficient knowledge about MVs [21,53,54], particularly for influenza vaccination [13,53].

Wilcox et al. [21] explained that concerns about safety for baby and mother were the key reasons for the lack of intention to have MVs, a finding that did not emerge strongly in the current study. However, similar to our research, Wilcox et al. found differences between influenza and pertussis vaccinations, with concerns about effectiveness, wrong season, and not believing vaccination is necessary or being advised against it by a staff member occurring more for influenza than pertussis. Wilcox et al. suggested framing the information towards the child’s safety and providing education for HCPs. However, we found that HCPs and women needed more information not only on the safety, but also on the benefit of antenatal influenza vaccination and the potential risk of influenza in pregnancy, of which very few participants were aware. While O’Shea et al. [53] found misconceptions about influenza vaccination as we did, they found the pertussis vaccination uptake was lower than the influenza vaccination in pregnant women, most likely because HCPs less frequently raised awareness of this vaccine.

Wilson et al. [39] found Black British Caribbean women distrusted the government regarding MVs, and family and friends were very influential in MV uptake. Neither of these findings emerged in our research, possibly reflecting the different ethnicities and life experiences for our women. Similar to our findings, O’Shea et al. [53] found family and friends were not particularly influential. However, similar to the research of Wilson et al. [39], we found HCPs often preferred/emphasised the maternal pertussis vaccination over influenza vaccination; some midwives mentioned MVs rather than actively recommending them; and some HCPs provided leaflets or suggested looking for online information, rather than discussing potential concerns, when women would prefer more discussion with the HCP.

Others have found HCP recommendation and/or endorsement of safety helps MV uptake [12,18,19,27], and that not all HCPs providing antenatal care provide this [12,13,19,30]. Certainly, we found the HCP important, particularly the midwife, but the general practice, pharmacy, and hospital HCPs also aided the uptake or sometimes missed opportunities to engage with women about MVs. Important within our findings was the strong influence of the underlying philosophy of choice in midwife’s messaging about MVs, contrasting with the other HCP groups. Similarly, in Canada, some midwives were concerned that a personal opinion or recommendation *“could compromise the principle of informed choice that is essential to the Canadian midwifery model of care…”* [20]. Australian research [30] found midwives tended not to personally recommend a patient receives a MV, and some struggled with a perceived tension between delivering public health messaging and respecting women’s choice [37]. Given the importance of women’s perceptions of their HCP’s support for one or both MVs, some midwives’ messaging to women to find their own information and decide for themselves might lead to an inference of ambivalence or doubt from the midwife regarding MVs, inadvertently affecting the uptake. With the reasonably straight-forward decisions women made about MVs, doubt may not prompt discussion with a HCP or reading information, but could instead simply result in a decision not to receive the vaccine as we saw with some women.

We found HCPs with recent training or experience with MVs appeared to be more confident with MV discussions and more proactive about MVs. Some midwives reported little undergraduate training about MVs (as others have found [30,36]), and general practitioners delegated vaccinations to practice nurses and therefore could be under-informed about MVs and not consider them in appointments with pregnant women. Pharmacists who did not provide vaccinations did not raise MVs with pregnant women. We recommend that a checklist be used for antenatal appointments to ensure MVs are discussed, and HCPs receive more training at an undergraduate level, possibly including how to administer MVs. To move HCPs from only raising awareness to actually providing the information that women need to help them to decide, HCPs’ training should include content about the women’s need for knowledge of the benefits and risks of MVs and the diseases they prevent, how women decide about MVs, appropriate messaging, and how to identify and manage vaccine hesitancy.

Our findings suggest that awareness of MVs needs to increase further in NZ, and this should be aided by early presentation to the midwife and general practitioners mentioning MVs at their antenatal appointments. HCPs in hospital who see pregnant women need to be well informed and understand the importance of their role in raising awareness of MVs. Having all pharmacies and hospital clinics seeing pregnant women provide vaccinations would increase the likelihood of both of these HCPs raising the awareness of pregnant women about MVs and offering them at that time.

MV uptake is aided by provision during antenatal appointments [26]. A discussion with an active offer of a vaccine thus making delivery as easy as possible for the woman could help the uptake, particularly where there were barriers to receiving the vaccine (e.g., other priorities). This may not be possible for midwives working outside of hospitals, but there were opportunities missed in other environments where MVs should have been available. It would help if all pharmacies provided vaccinations. The LMC could also offer to add MVs to a prescription at the relevant time point to help to remind the woman and pharmacist that the MV could be delivered while waiting for the rest of the prescription.

### 4.3. Strengths and Weaknesses

This study’s strengths include the large number of interviews and diversity of those interviewed, as the sample included women, midwives, general practice staff (doctors, nurses, and a practice manager), and pharmacists, as well as the purposive sampling that ensured that a range of experiences and views for each group were captured.

We included Māori and high needs areas where MV uptake is particularly low [16] and learnings could be greater. However, we only included one Pacific woman, despite the need to understand the influences on vaccine uptake for Pacific women.

Most women participants were recruited in pharmacy, so women not attending pharmacies were unlikely to be interviewed. Only one woman was under 20 years of age, an age group associated with low MV uptake [17]. Only three of the eighteen women participants had not received any MVs. Interviewing more women who had not received MVs, more young women, and a wider range of ethnicities would likely have been informative. We were reliant on participants’ recall, and some may have forgotten messages they had received around MVs. We did not confirm with medical records the woman’s recall of whether they had one or both MVs.

We did not aim for data saturation, but rather breadth of participants. As qualitative research, our findings cannot be generalised to all women or HCPs.

We took a systematic approach to the coding and analysis, and used sceptical review from the other authors; however, there remains a risk of bias with one person coding the data and conducting the analyses.

### 4.4. Future Research

Further exploration of HCP messaging and the perceptions of such messages from women regarding MVs would be helpful, for example, through video consultations with HCPs followed by an interview with the woman. This would help to show what form of messaging works the best, what women obtain from the messages provided, and how messages could be improved. It could also confirm or deny the possibility that the midwife’s careful support of women’s choice with MVs could inadvertently cause some women to doubt the benefit or safety of MVs, and/or lead her to believe that the midwife was not supportive of MVs. Research could also explore the effect of interventions with HCPs on the messaging provided to women and resulting in MV uptake. Within this same research project, we evaluated an intervention of funded availability of the maternal pertussis vaccination through pharmacies [35] and Māori women’s journey for maternal vaccination (soon to be published). Further work is needed to understand the journey for Pacific women.

Further qualitative research could focus solely on women not receiving MVs and on which messages would help them to decide to have a MV.

## 5. Conclusions

There appears to be a wide range of influences on women’s uptake of MVs and on HCPs’ recommendations for MVs. To increase the uptake of MVs, a multi-pronged approach is likely to be necessary. The role of HCPs seems vital. Encouraging early engagement with antenatal care should help to provide sufficient time for HCPs to inform women about MVs. All HCPs in the community and in the hospital who see pregnant women should be well informed about MVs and encouraged to provide clear information about MVs as well as offer and administer MVs where possible. Advising women about MVs should include discussing the benefits (particularly for the baby) and risks of not having MVs. HCPs should encourage women to ask questions, explore and address any concerns by women about MVs, and ideally have the vaccine offered on the spot when seeing a HCP. HCPs need to be well informed about how to communicate this information effectively such that it does not create doubt about MVs and how they can help to identify and correct misinformation the woman may have.

## Figures and Tables

**Table 1:** Characteristics of the enrolled and interviewed women (*n* = 18).

Variable	Māori or Pacific Women *n* = 10	Women Who Identified with Neither Māori nor Pacific Ethnicity *n* = 8
Identified ethnicity	9 Māori and 1 Cook Island Māori.	4 NZ European; 3 South African European; 1 Chinese (born in China).
Rural/urban	6/4.	2/6.
Age	18–31 years.	23–37 years.
Number ≤ 25 years	5	1
Number of weeks’ gestation or age of infant when interviewed	1 was 22 weeks’ gestation and 4 were 31–39 weeks’ gestation. 5 had infants aged 5 weeks to 4 months old.	3 women were 34–36 weeks’ gestation. 4 had infants 1 week to 4.5 months old; 1 had a 12-month-old infant.
Number of children	2 women had no other children, 3 had 1 other child, 1 had 2 other children, 1 had 3 other children, and 3 had 4 other children.	3 women had no other children; 4 had 1 other child, and 1 had 2 other children.
Lead maternity carer	All used a midwife.	All used a midwife.
First presentation to their midwife	5 women first saw their midwife at 4–7 weeks’ gestation; 2 at 12–15 weeks, and 3 at 25–27 weeks.	All women first presented to the midwife at 4–10 weeks’ gestation.
Received no MVs	2	1
Received both MVs (or planned to)	4 *	2
Received pertussis vaccine only	4	4
Received influenza vaccine only	0	1
Location of MVs	Pharmacy: 3. General Practice: 4 *. Hospital: 1.	Pharmacy: 6. General practice: 1. Hospital: 0

MV = Maternal Vaccination. * One woman planned to receive both MVs from her general practice.

**Table 2:** Characteristics of the enrolled and interviewed health care providers (*n* = 35).

Variable	Community Pharmacists (*n* = 12)	Midwives (*n* = 11)	General Practice Staff (*n* = 12)
Staff mix	10 (including 3 pharmacy owners) were trained vaccinators providing vaccinations. 2 were pharmacy owners in pharmacies that did not provide vaccinations, but 1 of these was a trained vaccinator and owned other pharmacies where vaccinations occurred.	1 worked at a hospital, 10 worked as Lead Maternity Carers (LMCs), 3 of whom had recent or current hospital or district health board experience.	4 general practitioners (2 practice owners), 7 practice nurses, 1 practice manager.
Rural/urban	7/5.	7/4.	8/4.
Practice details	6 worked in high needs areas, 2 were in higher socio-economic areas, the rest had a mixed socio-economic clientele.	2 described their area served as high socio-economic, the rest were in low socioeconomic or mixed socio-economic areas. The Māori midwives tended to have mainly Māori clients.	All worked in practices with a large proportion of high deprivation, with mixed high and low deprivation, and/or high Māori patient load. 2 worked at a Māori healthcare provider.
Work hours	11 worked full time and 1 worked part-time.	All worked full-time or close to it. A total of 2 worked around 60 h per week or more.	7 were full-time, 4 part-time and 1 unknown.
Identified ethnicity	6 NZ European, 3 Chinese/Asian), 1 Māori, 1 Fiji Indian, and 1 Middle Eastern.	5 Māori or part Māori, 4 NZ European, 1 British, and 1 Asian.	8 NZ European, 1 South Asian, 3 Māori or NZ European/Māori.
Experience	4 had 1–4 years’ experience, 5 had 9–18 years’ experience, and 3 had 30–40 years’ experience.	3 had 1–5 years’ experience, 4 had 7–15 years and 4 had 20–30 years’ experience.	2 had 2–5 years’ experience, 4 had 9–16 years’ experience, and 6 had 25–31 years’ experience.
Gender	8 female and 4 male.	All female.	All female.

## Data Availability

Owing to ethical and privacy issues, data are not available for sharing.

## Supplementary Materials

The following are available online at https://www.mdpi.com/article/10.3390/vaccines10010076/s1, Supplementary File S1: Qualitative Questionnaires—Women pregnant or who have recently had a baby and Health Care Providers.
