# Supplementary material for: Influences on Pregnant Women’s and Health Care Professionals’ Behaviour Regarding Maternal Vaccinations: A Qualitative Interview Study
Source: Vaccines (Basel). 2022 Jan 4;10(1):76. doi: 10.3390/vaccines10010076 (PMC8779619; doi:10.3390/vaccines10010076)
Supplement: Supplementary file 1 [file vaccines-10-00076-s001.zip › vaccines-1476181-supplementary.pdf]

Supplementary File S1: Qualitative Questionnaire – Women pregnant or who have recently had a baby

A conversational approach will be used with the following questions/topics as a guide; deviation is allowed if appropropriate.

Demographics: age, ethnicity, number of children, living arrangements [ask how many people usually live in the house where they live], where they live (suburb and town, etc). How many weeks pregnant they are currently, or how old their baby is (if post-delivery).

Which LMC was used for their pregnancy – midwife in the community, midwife at the hospital, GP, obstetrician/gynaecologist. How many months pregnant were they when the LMC was first seen.

What do you know about whooping cough in babies?

What do you know about influenza in pregnancy? [if necessary probe: did they know that influenza is more dangerous in pregnancy for the Mum, e.g. she will have a higher risk of hospitalisation.]

Do you know if you had your childhood vaccinations – e.g. vaccines at school or at the doctors?

What do you know about vaccines being given in pregnancy? Probe: what did you hear? [important] Where did you hear it? [probe on whooping cough/pertussis and influenza] What did different people tell you?

What do you think about the idea of vaccines in pregnancy?

Did you yourself have any vaccinations given to you during your pregnancy?

If vaccinated:

What vaccination/s were given? [prompt if necessary, influenza, pertussis]

Where did you get the vaccinations? [GP, practice nurse, midwife, hospital, pharmacist, work, other] Why did you go there for it?

When did you have the vaccinations? [how many weeks were you - influenza, pertussis]

Tell me about your decision to get a vaccination [probe, why did you, who influenced you, how did you know about it? Did you look up information on the internet or anywhere else?]

Who did you discuss it with?

Where did you find out about it? [probe: hospital, midwife, GP, pharmacy, poster, information sheet, family, friends, antenatal class, hapū wananga, facebook, other? Probe also how: e.g. was it suggested, did they see written material, a video?]

If written information or social media or other media was used, where did you see or hear this? [probe also for how much it influenced their decision]

What was the most important thing that helped you decide to have the vaccination?

Did you have any concerns about the vaccine? [if so, what were they, where did they come from (e.g. friends, health professionals, websites), and how were they overcome?]

What was your experience of getting the vaccine?

Probe about pertussis primarily but also influenza vaccination.

If not vaccinated with any vaccination during pregnancy, or if one vaccine was given but not the other:

Tell me about that. [probe: what were the reasons why they had not had a vaccination?]

Had anyone discussed the vaccine/s with you, [probe midwife, GP, nurse in the GP, nurse at the hospital, pharmacy staff, friends, family, other?] if so who discussed it with you. What did they say, and how did that influence your decision?

Was there anything that made it difficult for you to get the vaccine? [probe if necessary: expecting a cost, transport difficulties, booking an appointment, difficulty finding the time, pressure/persuasion from others not to be vaccinated, lack of child care]

Did you have any concerns about the vaccine? [if so, what were they? What was the most important concern? Where do you hear about that concern?, e.g. facebook, friends, family]

Did you see any information about getting vaccinated [posters, facebook, leaflets, etc, video] What did you think about that? Did it influence you and if so in what way?

For all:

What do you think about vaccination generally?

Have you had your children vaccinated?

Do you know where pregnant women can get free vaccines for influenza and pertussis from? [prompt general practice, hospitals, pharmacies]

What could make it easier for women like you to get vaccinated during pregnancy?

Health professionals

Qualitative Questionnaire – Healthcare providers

Probe as appropriate throughout on pertussis and/or influenza. Conversational interview guide, deviate as needed.

Demographics: Number of years practising as a health care professional. Gender, ethnicity. Where do they practice? How long have they practised there? How many hours per week do they work?

What role if any do you have in vaccinations? [if necessary probe on the following do vaccinations, recommend vaccinations, provide information, do reminders for vaccinations by phone/mail?]

For those administering vaccinations:

How long have you administered vaccinations?

How many vaccinations would you do per week or per month [specify which]?

How many vaccinations of pregnant women would you have administered in the last month?

For those not administering vaccinations:

Why do you not administer vaccinations? [probe logistics, funding]

For all:

What do you think about the idea of giving vaccinations in pregnancy?

Tell me about your understanding of vaccination recommendations in pregnancy? [probe: pertussis and influenza if necessary, recommended timing, funding, and where they are available from in Waikato]

Where do you recommend women get their vaccination? Why?

Why do you think some women do not get vaccinations in pregnancy?

What do you think helps women get vaccinations in pregnancy?

Are there any particular groups who you think are more likely to get vaccinations in pregnancy? If so, why are they?

Are there any particular groups who you think are less likely to get vaccinations in pregnancy? If so, why not?

What do you think needs to be done to increase the number of women getting vaccinations in pregnancy?

What (if anything) have you or your practice done to help women get vaccinated in pregnancy? [probe: pamphlets, posters, recommendations, telephone reminders, no wait vaccines. How well do you think that is working?]

What makes it harder to recommend vaccinations in pregnancy [probe time, knowledge, remembering]

For providers of vaccines:

How does it work for your practice/pharmacy/you personally to provide vaccinations to pregnant women?

How many pregnant women per week or per month would be getting vaccinated at your pharmacy/would you have vaccinated [general practice/midwife]?

What challenges do you/your practice experience with vaccines? [probe if necessary: staff availability, fridge space, out of stocks, insufficient funding, too busy]

General practice: How long does it take to get an appointment in [your practice/pharmacy, or in local providers, as applicable] for a vaccination? If appropriate: What days and times can people get vaccines?

What helps you provide vaccinations in your practice/pharmacy?

For pharmacy:

Does your pharmacy provide vaccines or not? Why/why not?

What other barriers are there for pharmacy generally to providing the vaccinations? [if necessary probe stock, fridge, staff aspects, busyness, inadequate funding, accessing training, patients do not want it, no interest or think it is not appropriate to pharmacy, no consultation room, or consultation room used for other reasons, concern about liability, do not like vaccines, paperwork eg SOPs, set up costs, local GP concern]

What other enablers are there for pharmacy generally to providing the vaccinations? [probe: funding, help from pharmacy organisations, encouragement from other health providers or the DHB]

Which of your staff members know about the vaccinations?

Tell me about how most pregnant women present for vaccinations [e.g. patient request, suggested in the pharmacy, get an idea of the proportion of each]
